# Altered Levels of Serum Zinc and Cadmium in Patients with Chronic Vesiculobullous Hand and Feet Dermatitis

**DOI:** 10.1155/2016/3284937

**Published:** 2016-04-06

**Authors:** Swastika Suvirya, Alpna Thakur, S. S. Pandey, S. K. Tripathi, Durgesh Kumar Dwivedi

**Affiliations:** ^1^Department of Dermatology, King George Medical University, Lucknow, Uttar Pradesh 226003, India; ^2^Department of Dermatology, IMS, Banaras Hindu University, Varanasi, Uttar Pradesh 221005, India; ^3^Department of Forensic Medicine, IMS, Banaras Hindu University, Varanasi, Uttar Pradesh 221005, India; ^4^Department of Radiodiagnosis, King George Medical University, Lucknow, Uttar Pradesh 226003, India

## Abstract

Micronutrients serve many important functions in our body and altered levels of heavy and trace metals are associated with cutaneous and systemic disorders. Vesicular palmoplantar eczema is an entity whose etiopathogenesis is a mystery. In this prospective case-noncase study blood levels of Zinc and Cadmium in 37 patients of chronic vesiculobullous hand dermatitis were estimated and compared with 40 noncases with similar age and gender distributions. Low serum Zinc levels were found in patients as compared to noncases. The mean difference of serum Zinc between the case and noncase groups was 27.26; the mean value of serum Zinc between the two groups was statistically significant (*p* < 0.0001). However, elevated Cadmium levels were detected in only 5 patients and in none of the noncases. The mean concentration of serum Cadmium was 2.32 ± 0.38 *μ*g/dL, with a range of 1.90–2.80 *μ*g/dL for the five cases in whom Cadmium was detected. Various toxic and trace metals can interact by influencing each other's absorption, retention, distribution, and bioavailability in the body. The clinical significance of this finding lies in the possible beneficial role of Zinc supplementation in the therapy of chronic vesiculobullous hand dermatitis.

## 1. Introduction

Vesicular palmoplantar eczema is characterised clinically by small to large blisters in the hands and feet and histologically by spongiosis. Its etiopathogenesis remains obscure. It is divided into 4 categories: pompholyx, chronic vesiculobullous hand dermatitis, hyperkeratotic hand dermatitis, and id reactions [1]. However, in many dermatology textbooks, this classification is not followed and vesicular eczema of hands and feet, dyshidrotic eczema, and pompholyx are synonymous and used interchangeably [2].

Chronic vesiculobullous hand dermatitis is difficult to manage because of its relapsing course. Contact allergy, ingestion of metals, atopy, psychological stress, and hot weather are all postulated to play a role in its pathogenesis. The clinical presentation includes small 1 to 2 mm vesicles filled with clear fluid localizing to the lateral aspects of the fingers, palms, and soles. With time, the clinical appearance may evolve and subsequently appear more fissured and hyperkeratotic [1].

Zinc is involved in numerous aspects of cellular metabolism. It is required for the catalytic activity of more than 200 enzymes and it plays a role in immune function, wound healing, protein synthesis, DNA synthesis, and cell division. Zinc has antioxidant properties, which may protect against accelerated aging and helps speed up the healing process after an injury [3].

Low levels of Zinc have been found in hair and erythrocytes of patients of atopic dermatitis (AD). Zinc supplementation has led to improvement in scores assessing severity in AD [4]. Cadmium is a toxic heavy metal found in low concentrations in blood or is generally undetectable [5]. Elevated levels are found in smokers and persons residing in industrial areas [6]. Altered Zinc/Cadmium ratios have been found in industrial workers and also in certain disorders like prostate cancer [7], decreased renal function [8], hypertension, and coronary artery disease as various toxic and trace metals can interact by influencing each other's absorption, retention, distribution, and bioavailability in the body. Toxicity of Cadmium has been found to be dependent on disturbance in Zinc metabolism, earning the name of “antimetabolite” of Zinc [7]. Thus, there is a need to estimate the levels of serum Zinc and Cadmium in patients with chronic vesiculobullous hand dermatitis.

## 2. Methods

**Figure 1:**
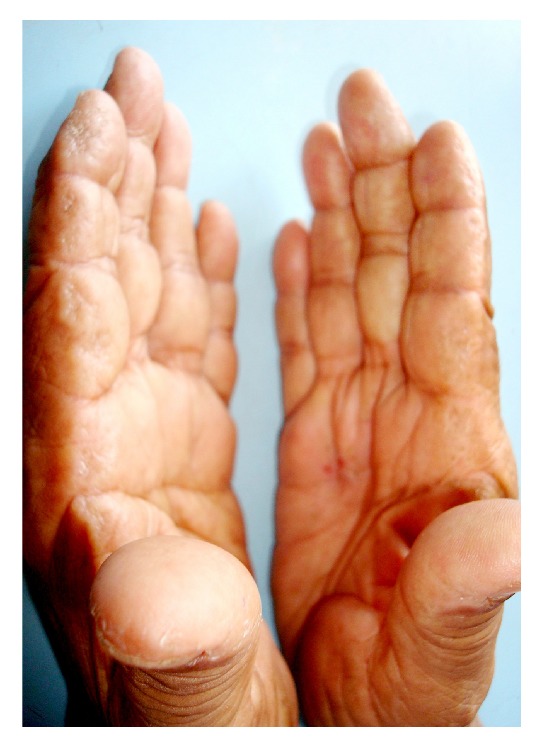
Multiple tiny vesicles involving lateral aspect of fingers.

**Figure 2:**
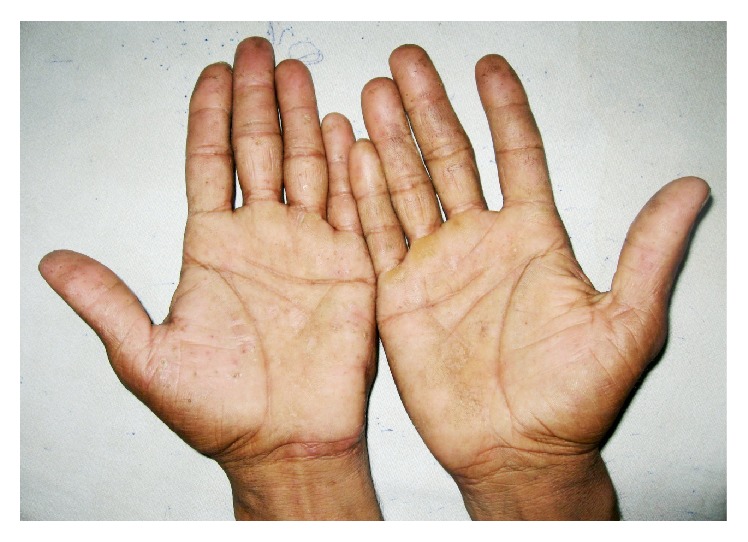
Multiple vesicles symmetrically distributed over both palms.

**Figure 3:**
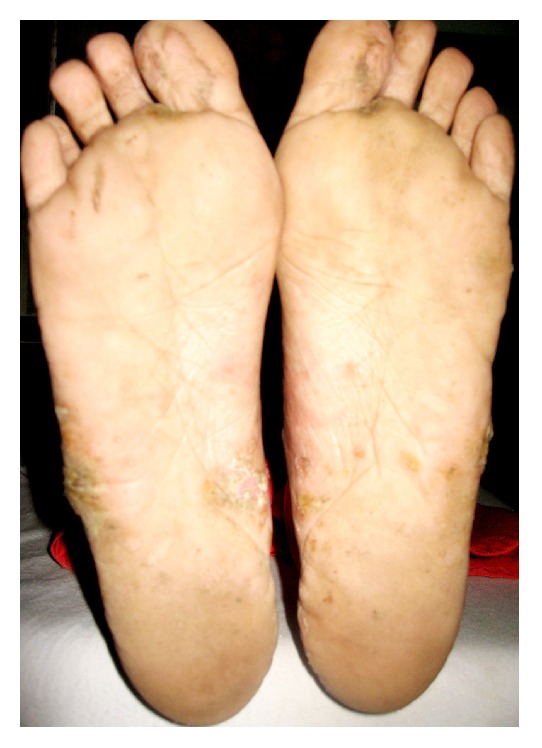
Scaly, crusted plaque involving both feet.

The study was a prospective case-noncase study carried out in the dermatology outpatient department for one-year period. The protocol was approved by the institutional ethics committee. The study population included 37 cases with chronic vesiculobullous hand dermatitis and 40 age and sex similar noncases. Inclusion criteria were duration of vesicular eczema for more than 6 weeks, bilateral lesions on palms, and/or soles (Figures 1, 2, and 3). Patients suffering from generalized diseases, having lesions on other parts of body, and cases of allergic contact dermatitis were excluded from the study. Skin scraping for fungus and patch testing with Indian Standard Patch Test Battery approved by the Contact and Occupational Dermatosis Forum of India (CODFI) were done to rule out other types of hand eczema, like contact dermatitis and id reactions. Forty noncases with similar age and gender distribution were taken who were suffering from benign dermatological disorders like acne (*n* = 14), melasma (*n* = 9), pityriasis versicolor (*n* = 7), tuberculoid Hansen's disease (*n* = 4), and borderline tuberculoid Hansen's disease (*n* = 6). The noncases taken in the study did not have any generalized diseases and hand and foot eczema on examination nor had any history of eczema.

About 5 mL of blood was collected in ethylenediaminetetraacetate vial and stored in the refrigerator immediately at 4°C and later transported to the laboratory in Department of Forensic Medicine. The concentration of Zinc and Cadmium was estimated by Atomic Absorption Flame Spectrophotometer (Model number SL-194; ELICO), which is a double beam spectrophotometer. Data was entered into a MS EXCEL database and analyzed using SPSS version 10.00 for Windows. Data were described using appropriate summary measures. The statistical tests used in the study were unpaired *t*-test and chi-square test. A *p* value <0.05 was considered significant.

## 3. Results

**Figure 4:**
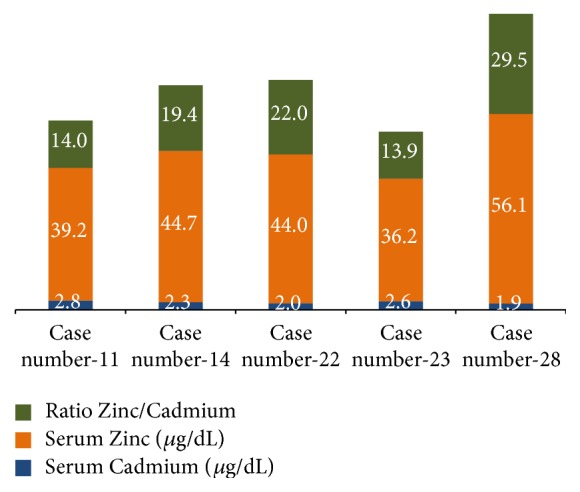
Serum Zinc concentration in cases in whom Cadmium was detected.

Table 1 shows history of signs, symptoms, and clinical profile of cases. The comparison of epidemiological profile of cases and noncases is shown in Table 2.  Table 3 shows comparison of serum level of Zinc and Cadmium between cases and noncases.  Figure 4 shows ratio of serum Zinc and Cadmium in cases where both metals were detected.

Zinc was detected in sera of all the cases and noncases. The range of serum Zinc in cases was 17.20–57.90 *μ*g/dL and in noncases was 42.00–102.20 *μ*g/dL. The mean of serum Zinc in 37 cases was 39.95 ± 9.43 *μ*g/dL, whereas, for the 40 in noncase group, the mean of serum Zinc was 67.21 ± 13.77 *μ*g/dL. The mean difference of serum Zinc between the case and noncase groups was 27.26. This difference was found to be statistically significant (*p* < 0.0001). The 95% confidence interval of the difference between serum concentrations was 21.86 to 32.66.

There was no statistically significant difference in concentration of serum Zinc with regard to the place of residence (urban versus rural; *t*-test, all *p* > 0.14), dietary habits (vegetarian versus nonvegetarian; *t*-test, all *p* > 0.47), age groups (*t*-test, all *p* > 0.014), or gender (*t* = 0.15, *p* = 0.88). This is similar to the observation by Hashim et al. that concentration of serum Zinc does not vary with age or gender [9].

Cadmium in blood could be detected in only 5 cases, with the mean concentration being 2.32 ± 0.38 *μ*g/dL and in the range 1.90–2.80 *μ*g/dL. Cadmium was below detectable levels (BDL) in rest of 32 cases and in all the 40 noncases. The BDL for Cadmium was 0.009 ppm for the instrument.

## 4. Discussion

A number of etiologic factors have been associated with vesicular palmoplantar eczema, including atopy, contact allergy, psychological stress, and hot weather. Crosti and Lodi found that patients with pompholyx had no sweat gland dysfunction or dyshidrosis as was previously thought, although hyperhidrosis could be an exacerbating factor [10]. In another study by Lodi et al. of 104 patients, they found that hyperhidrosis was the exacerbating factor in 37% patients [11]. In our study, only 1 out of 37 (2.7%) cases had palmoplantar hyperhidrosis as the exacerbating factor.

In a study by Lodi et al. of 104 patients of pompholyx, personal or family history of atopy was found in 50% of the patients versus 11.5% of noncases [11]. Others have found no association between atopy and pompholyx [12–14]. In our study, 3 patients (8.1%) had history of similar complaints in first-degree relative, while 34 (91.8%) had no such history. Genetic predisposition to vesicular palmoplantar eczema has been noted [15].

Clinical presentation of chronic vesiculobullous hand dermatitis includes small 1-2 mm vesicles filled with clear fluid, most commonly seen on the lateral aspect of fingers, palms, and soles. Similarly, lesions of pompholyx develop symmetrically on the palms and/or soles in a typical case. In 80% of patients of pompholyx only the hands are involved while in 10% of the patients hands and feet both are involved. Rubbing and inappropriate treatment may produce secondary eczematous changes. With time, the lesions become fissured and hyperkeratotic. Secondary infection with pustule formation is not uncommon in patients of pompholyx [1, 2].

The essentiality of Zinc for humans was recognized in 1961 [16]. Zinc deficiency is common in many developing nations. Diets based on cereals and legumes and poor in animal products make it difficult to meet the Zinc requirements because their high phytate content reduces the bioavailability of Zinc [17]. Zinc has been found to be low in children with atopic dermatitis [4]. Epidermal Zinc concentration was found to be low in patients with dermatitis herpetiformis, acne, psoriasis, and Darier's disease [18]. Measurement of plasma Zinc in psoriasis has resulted in conflicting data with both reduced and normal levels being reported [19]. Serum Zinc levels were found to be significantly low in patients of psoriasis, acne vulgaris, and leprosy [20].

The range of serum Zinc in our study cases was 17.20–57.90 *μ*g/dL and in noncase group, it was 42.00–102.20 *μ*g/dL. With the normal serum and plasma concentration of Zinc ranging between 70 and 100 *μ*g/dL [9], cases in our study were clearly Zinc deficient.

Studies on mice fed on a Zinc deficient diet have shown that Zinc deficiency leads to development of more severe skin lesions as compared to controls, with higher transepidermal water loss in deficient mice. Levels of IgE, interferon *γ* (INF*γ*), and interleukin-13 (IL-13) were also raised in Zinc deficient mice [21]. In humans, Zinc deficiency has been reported to cause an imbalance between Th1 and Th2 cells: the Th1 function is decreased, while Th2 function is not affected, with increase in the production of IFN*γ*, IL-2, and tumour necrosis factor *α* (TNF*α*) [22]. Studies done with cell culture models show decrease in NF-*κ*B activation with subsequent decrease in inflammatory cytokine production (like TNF*α*, IL-1*β*, and IL-8). Thus, Zinc deficiency leads to increased Th2 cytokines and reduced anti-inflammatory effects, leading to possible eczema-like skin lesions [23]. This could also be a possible reason for chronic vesiculobullous hand dermatitis in our Zinc deficient cases.

Cadmium is a toxic heavy metal normally not detectable in blood, with normal levels <0.5 *μ*g/100 mL [5]. High concentrations are however found in smokers and persons exposed to industrial fumes [6]. Since it was detected in only 5 patients in our study, its role in pathogenesis seemed doubtful and was not analyzed further.

Till date, there is no study indicating that Zinc could play a role in the pathogenesis of patients with vesicular hand dermatitis. In the present study, serum Zinc was found to be significantly low and below the normal range in patients of vesicular palmoplantar eczema. Also we could not estimate the elements a second time when lesions had subsided. Exact role of Zinc in the pathogenesis of chronic vesiculobullous hand dermatitis needs to be studied further.

## 5. Conclusion

The present study points towards a possible, hitherto unknown, association of Zinc deficiency in patients of chronic recurrent vesicular hand dermatitis. The clinical significance of this finding lies in the possible beneficial role of Zinc supplementation in the therapy of palmoplantar vesicular eczema.

To confirm our findings regarding levels of Zinc and vesicular palmoplantar eczema, a large scale prospective study with a long follow-up and further analysis is needed. A detailed estimation of these metals at various stages of the disease is needed. This will help in providing proper insight into their role in the pathogenesis of vesicular palmoplantar eczema. Further, clinical therapeutic trials would also be needed to substantiate a role for Zinc supplementation in these patients.

## Figures and Tables

**Table 1:** Clinical profile in cases (*n* = 37).

Variables	Number of cases	%
*Duration of itching*		
<3 months	25	67.6
4 to <6 months	5	13.5
>6 months	5	13.5
No itching	2	5.4
*Intensity of itching*		
Mild	14	37.8
Moderate	19	51.4
Severe	2	5.4
*Diurnal variation in itching*		
More in daytime	14	37.8
More in night	3	8.1
No diurnal variation	18	48.6
*Duration of skin lesions*		
<3 months	27	73
4–6 months	5	13.5
>6 months	5	13.5
*Past history of recurrent disease*		
Yes	29	78.4
No	8	21.6
*Previous treatment taken*		
Allopathic	33	89.2
Ayurvedic	1	2.7
No treatment	3	8.1
*Personal history of atopy*		
Yes	1	2.7
No	36	97.3
*Family history of atopy*		
Yes	4	10.8
No	33	90.2
*Site of lesions*		
Palmar areas of hand	29	78.4
Sides of finger	37	100
Dorsum of hand	19	51.4
Dorsum of foot	3	8.1
Only on hands	30	81.1
Both hands and feet	7	18.9
*Morphology*		
Vesicular	25	67.6
Erosions	33	89.2
Crusting	12	32.4
Scaling	28	75.6
Pustules	4	10.8
*Pattern of lesions*		
Confluent	35	94.6
Discrete	14	37.8
Clustered	1	2.7

**Table 2:** Comparison of epidemiological profile of cases and noncases.

Variables^a^	Cases (*n* = 37)		Noncases (*n* = 40)		*p* value
	No	%	No	%	
*Sex*					
Male	26	70.3	26	65	0.62
Female	11	29.7	14	35	
*Religion*					
Hindu	34	91.9	36	90	0.45
Muslim	2	5.4	4	10	
Christian	1	2.7	0	0	
*Residence*					
Urban	9	24.3	15	37.5	0.21
Rural	28	75.7	25	62.5	
*Marital status*					
Married	29	78.4	29	72.5	0.55
Unmarried	8	21.6	11	27.5	
*Diet type*					
Vegetarian	14	37.8	16	40	0.85
Nonvegetarian	23	62.2	24	60	
*Occupation*					
Housewives	7	18.9	9	22.5	0.62
Farmers	4	10.8	7	17.5	
Students	8	21.6	12	40	
Businessmen	6	16.2	3	7.5	
Labourer	3	8.1	3	7.5	
Servicemen	6	16.2	4	10	
Others	3	8.1	3	7.5	

^a^Chi-square test used.

*p* value < 0.05 is significant.

**Table 3:** Comparison of serum Zinc and Cadmium levels between cases and noncases.

	Zinc		Cadmium	
	Cases	Noncases	Cases	Noncases
*N*	37	40	5	40
Range	17.20–57.90	42.00–102.20	1.90–2.80	BDL
Mean	39.95	67.21	2.32	NA
SD	9.43	13.77	0.38	NA
Mean difference	27.26		—	
95% CI of differences	21.86–32.66		1.84–2.79	
*p* value	*<0.001*		—	

Independent *t*-test used.

*p* value < 0.05 is significant.
